# *Petrocodon wenshanensis*, a new species of Gesneriaceae from southwestern China

**DOI:** 10.3897/phytokeys.157.39624

**Published:** 2020-08-26

**Authors:** Zheng-Long Li, Wei-Hua Qin, Fang Wen, De-Ming He, Xin Hong

**Affiliations:** 1 Anhui Provincial Engineering Laboratory of Wetland Ecosystem Protection and Restoration, School of Resources and Environmental Engineering, Anhui University, CN-230601, Hefei City, Anhui Province, China Anhui University Hefei China; 2 Nanjing Institute of Environmental Sciences, Ministry of Ecology and Environment of the People’s Republic of China, CN-210042, Nanjing City, Jiangsu Province, China Nanjing Institute of Environmental Sciences Nanjing China; 3 Guangxi Key Laboratory of Plant Conservation and Restoration Ecology in Karst Terrain, Guangxi Institute of Botany, Guangxi Institute of Botany, Guangxi Zhuang Autonomous Region and Chinese Academy of Sciences, CN-541006, Guilin City, Guangxi Zhuang Autonomous Region, China Guangxi Institute of Botany Guilin China; 4 The Gesneriad Conservation Center of China, Guilin Botanical Garden, Chinese Academy of Sciences, CN-541006 Guilin, Guangxi, China Guilin Botanical Garden, Chinese Academy of Sciences Guilin China; 5 Administration of Wenshan National Nature Reserve, CN-663000, Wenshan Zhuang and Miao Autonomous Prefecture, Yunnan Province, China Administration of Wenshan National Nature Reserve Wenshan China

**Keywords:** Didymocarpoideae, limestone flora, new taxa, Yunnan Province

## Abstract

A new species of *Petrocodon*, *P. wenshanensis* from Yunnan province of southwestern China, is described and illustrated here. *P. wenshanensis* morphologically closely resembles *P. jingxiensis* and *P. lithophilus*, but differs in vegetative and generative characters. Differences between the new species and others *Petrocodon* species occurring in Yunnan Province are also shown in the identification key.

## Introduction

*Petrocodon* Hance has recently been much expanded and is now one of the most morphologically variable genera in Asian Gesneriaceae (Weber et al. 2011), with a wide range of corolla and leaf morphology (Lu et al. 2017a). Yunnan is one of the world’s 34 most species-rich regions, with the highest biodiversity resources in China (Huang et al. 2011). However partly for natural and geographic reasons associated with Yunnan, there are still enormous species waiting to be discovered and further revised. Recently, four new Gesneriaceae species were reported here (Chen et al. 2014; Chen et al. 2019), so there are now a total of 6 species of this genus found in Yunnan Province: *Petrocodon ainsliifolius*, *P. lithophilus*, *P. viridescens*, *P. tenuitubus*, *P. coccineus* and *P. hispidus*.

During an expedition to prepare a National forest resources inventory in April 2016, one of the authors, De-Ming He discovered some plants without flowers in Funing County, Wenshan Zhuang and Miao Autonomous Prefecture, Yunnan Province, south-western China. Afterwards, the other authors collected specimens from the same cave when they were undertaking the field works in ecologically functional zones of Yunnan Province. On the basis of its habit (leaves in basal rosette), corolla shape (limb 2-lipped), number of fertile stamens (two), anthers (dorsifixed, coherent apically) and capsule dehiscence (loculicidal), it was identified as belonging to *Petrocodon* s.l. (Wang 1990, 1998; Weber et al. 2011). At the time, they improperly identified it as *P. jingxiensis* (Y. Liu, H.S. Gao & W.B. Xu) A. Weber & Mich. Möller (Weber et al. 2011) due to the similar characters of leaf morphologies. In order to study it thoroughly, particularly the floral morphology, it was cultivated during the past three years in the greenhouse of the Gesneriad Conservation Center of China, which is located in the Guangxi Institute of Botany. The specimens of the new species were deposited in IBK and ANU and living individuals were cultivated at the Gesneriad Conservation Center of China. All morphological characters were studied under dissecting microscopes and were described using the terminology proposed by Wang (1990), Wang et al. (1998). After consulting the monographs (Li and Wang 2005, Wei et al. 2010) and comparing the species with all other congeners described (i.e. Chen et al. 2014, Chen et al. 2019, Xu et al. 2014, Hong et al. 2014, Guo et al. 2016, Cen et al. 2017, Lu et al. 2017b, Zhang et al. 2018, Li et al. 2019) and specimens of Gesneriaceae deposited at IBSC, IBK, KUN, PE, US and VMN. We confirmed that it is a new species and hence we describe and illustrate it below as such. A morphological comparison between *P. wenshanensis* and its congeners is provided in Table 1 and the Key.

**Table 1.** Diagnostic character differences between *Petrocodon wenshanensis* and its close relatives: *P. jingxiensis*, *P. lithophilus*.

Character	*P. wenshanensis*	*P. lithophilus*	*P. jingxiensis*
**Leaf blade**	apex acute	apex rounded	apex obtuse or rounded
**Bracts**	glabrous inside	pubescent on both sides	pubescent on both sides
**Calyx**	glabrous inside	pubescent inside	puberulent inside
**Corolla**	purple	light greenish yellow	purple
**Corolla tube**	gradually dilated and bent towards the throat	thin tubular	slender
**Corolla lobes**	apex acute, margin erosulate near the apex	apex acute, margin entire	apex round, margin entire
**Filaments**	strongly geniculate at the middle, glabrous	straight, glabrous	straight, puberulent

## Key to the species of the *Petrocodon* (Gesneriaceae) occurring in Yunnan Province, China

**Table:** 

1	Adaxial corolla lip 4-fided, the abaxial integrate	**2**
–	Adaxial corolla lip 2-fided, the abaxial 3-fided	**3**
2	Flower red; leaf blades elliptic	***P. coccineus***
–	Flower greenish; leaf blade round	***P. viridescens***
3	Flowers greenish	***P. lithophilus***
–	Flowers purple	**4**
4	bracts 3, corolla tubes curved	***P. tenuitubus***
–	bracts 2, corolla tubes strict	**5**
5	Corolla lobes acuminate, not reflexed	***P. wenshansnsis***
–	Corolla lobes caudate, reflexed	**6**
6	Leaf margin shallowly crenulate, apex rounded	***P. hispidus***
–	Leaf margin entire, apex acute	***P. ainsliifolius***

## Taxonomic treatment

### Petrocodon wenshanensis

Taxon classificationPlantaeLamialesGesneriaceae

Xin Hong, W.H. Qin & F. Wen sp. nov.

2692BEB3-21EC-59CA-B9A1-2047B415F37C

urn:lsid:ipni.org:names:77211195-1

Figure 1

#### Diagnosis.

The new species is vegetatively similar to *P. jingxiensis*, it differs from the latter in having acute apex of leaf blade (*vs.* obtuse or rounded), bracts and calyx glabrous inside (*vs.* pubescent), corolla tubes gradually dilated and bent towards the throat (*vs.* slender tubular), and lobes margin erosulate (*vs.* entire), filaments strongly geniculate at the middle, glabrous (*vs.* straight, puberulent). And it is also morphologically similar to *P. lithophilus*, but differs by its apex of leaf blade acute (*vs.* rounded), bracts and calyx glabrous inside (*vs.* pubescent), corolla purple (*vs.* light greenish yellow), tubes gradually dilated and bent towards the throat (*vs.* thin tubular) and lobes margin erosulate (*vs.* entire), filaments strongly geniculate at the middle (*vs.* straight).

**Figure 1.**
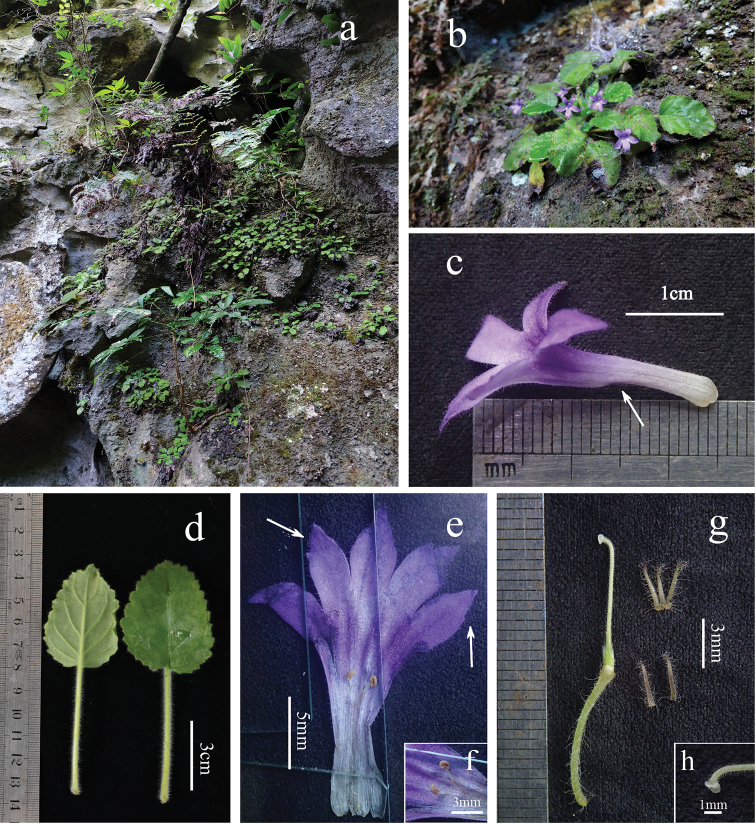
*Petrocodon wenshanensis* Xin Hong, W.H. Qin & D.M. He. **A** Habitat **B** habit when in flower **C** lateral view of corolla (arrow indicates the bent of the throat) **D** leaf blade **E** opened corolla (arrow indicates margin of corolla lobes erosulate near the apex) **F** stamens **G** pistil without corolla, showing calyx segments, inside glabrous (above), outside strigose (below) **H** stamens.

#### Type.

China. Guangxi Province, cultivated in the nursery of Gesneriad Conservation Center of China (GCCC), introduced from Yunnan Province: Muyang Town, Funing County, Wenshan Zhuang and Miao Autonomous Prefecture, 23°33'N, 105°28'E, 1,360 m a.s.l., growing in rocky crevices at the mouth of a karst cave. 14 June 2019, flowering, WF170807-06 (holotype: IBK; isotype: AHU).

#### Description.

Perennial herbs. Rhizomatous stem subterete, 30–75 mm long, 6–10 mm in diameter. Leaves basal, opposite and congested at rhizome apex; petiole 1.5–4.5 cm long, densely pubescent. Leaf blade chartaceous, ovate, 3–6 × 2–4 cm, apex acute, base broadly cuneiform or shallowly cordate, slightly oblique, margin crenulate, densely pubescent on both surfaces, lateral veins ca. 4 pairs on each side of midrib, concave adaxially, prominent abaxially. Cymes 1–3, axillary, 1–2-branched, 1–3(–6)-flowered; peduncles 4–6 cm long, densely glandular puberulent and sparsely strigillose; bracts 2, opposite, linear, ca. 5 × 0.8 mm, outside densely strigose, inside glabrous. Pedicels 2–8 mm long, glandular and eglandular pubescent. Calyx 5-lobed from base, segments narrowly lanceolate-linear, equal, ca. 5 × 0.9 mm, outside strigose, inside glabrous. Corolla zygomorphic, purple, 2.5–3.0 cm long, glandular and eglandular pubescent on both sides; corolla tubes infundibula-form, slender, gradually dilated and bent towards the throat; 17–19 mm long, 4–6 mm in diameter at the mouth, 2–2.5 mm in diameter at the base; limb distinctly 2-lipped; adaxial lip 2-lobed to the base, lobes ca. 8 mm long, orbicular triangular, apex acute, margin erosulate only near the apex; abaxial lip 3-lobed to the base, lobes 8–9 mm long, subequal, oblong, apex acute, margin erosulate only near the apex to nearly entire. Stamens 2, adnate ca. 10 mm above the corolla base, glabrous; filaments 2–3.5 mm long, strongly geniculate at the middle; anthers yellow, nearly reniform, ca. 1 mm, fused by their entire adaxial surfaces, dehiscing longitudinally; staminodes 3, linear, 0.5–0.7 mm long, adnate ca. 8 mm above the corolla base. Disc ring-like, ca. 0.7 mm high, with repand margin. Pistil 1.3–1.5 cm long; ovary ca. 2 × 1 mm, pilose; style 11–13 mm long, densely pilose, stigmas 2, ovate, ca. 0.6 mm long, lobes ca. 0.4 mm long. Capsule narrowly elliptic, dehiscing loculicidally into 2 valves.

#### Etymology.

The specific epithet is derived from the type locality, Wenshan Zhuang and Miao Autonomous Prefecture, Yunnan Province, China.

#### Vernacular name.

Wén Shān Shí Shān Jù Tái (Chinese pronunciation); 文山石山苣苔 (Chinese name).

#### Distribution and habitat.

**Figure 2.**
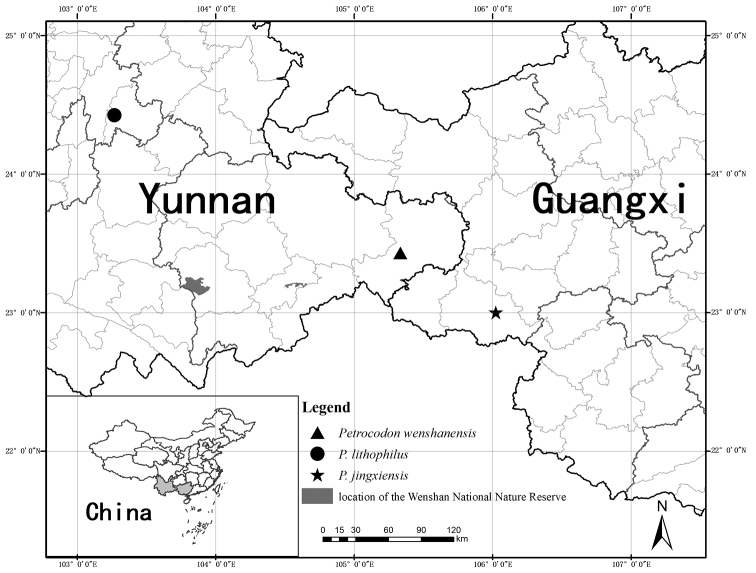
Distribution of *Petrocodon wenshanensis* (▲) and its similar species: *P. jingxiensis* (★), *P. lithophilus* (●), and the location of the Wenshan National Nature Reserve.

The new species has so far been found only in the type locality, Muyang Town, Funing County, Wenshan Zhuang and Miao Autonomous Prefecture, Yunnan Province, China. The geographical distributions of *Petrocodon wenshanensis* and its similar species are identified in Figure 2. The forest where *P. wenshanensis* occurs is monsoon evergreen broad-leaved forest. The average temperature of Funing County is about 19.8 °C and the average annual precipitation is over 1103 mm. Flowering is from June to August.

#### Provisional conservation status.

The landform of the type locality is karst topography, and the new species seems locally abundant in the limestone cave, the type locality. But all individuals grow on moist and shaded rocky faces on the cliff at the mouth of a karst cave. After carefully estimating and counting, the type population consists of approx. 8000 mature individuals. Although this type of cave is near the Wenshan Nature Reserve, it is also very close to the local village, so is excluded from the range of the reserve and is not protected by the reserve’s law. Hence it is easily disturbed by human activities (i.e reclamation and quarrying activities). Based on five careful field investigations over recent years, there has been no significant change in the number of individuals Thus, following the IUCN Red List Categories and Criteria (IUCN 2019), it is temporarily assessed as Critically Endangered [CR B2ab (iii)].

## Supplementary Material

XML Treatment for Petrocodon wenshanensis
